# AML treatment in older adults

**DOI:** 10.18632/aging.100816

**Published:** 2015-09-27

**Authors:** Alison Sehgal, Michael Boyiadzis

**Affiliations:** University of Pittsburgh Cancer Institute, University of Pittsburgh School of Medicine, Pittsburgh, PA 15232, USA

**Keywords:** acute myeloid leukemia, intensive chemotherapy, outcomes, novel therapies

Acute myeloid leukemia (AML) is an aggressive hematologic malignancy that affects people of all ages but becomes more common with increasing age. The median age at diagnosis is 67 years, and nearly 1/3 of patients are ≥75 years old at diagnosis [1]. However, successful treatment of AML in older patients (typically defined as >60 years in those with AML) presents a significant challenge due to poor tolerance of the standard chemotherapy and adverse biological features of the leukemic cells compared to younger patients. Older patients are more likely to have adverse cytogenetic features, antecedent hematologic disorders that predispose to chemotherapy resistance, and a higher expression of multidrug resistance genes. Furthermore, their increased frequency of comorbidities and decreased performance status at the time of diagnosis leads to poorer tolerance to therapy compared to younger patients. Combined, these features lead to a 5-year leukemia free survival of only 8.5% older adults (age 65-74) compared to 39% in those under age 65 [2].

Traditional therapy for patients with AML involves intensive chemotherapy, usually involving an anthracycline combined with cytarabine or other cytotoxic drugs. Although older patients tend to have worse outcomes with this therapy compared to younger patients, intensive induction therapy appears to have a small but real survival advantage compared to supportive care alone in older patients [3]. As such, an individualized approach to offering intensive therapy to older adults is important to choose those older patients who may obtain a survival benefit with minimal toxicity. Several predictive scores have been investigated as decision criteria for older AML patients who are treated with intensive chemotherapy [4]. Cytogenetics, performance status, age, white blood cell count at diagnosis and organ dysfunction are some of the variables identified as related to prognosis in different scoring systems. Using these scoring systems, patients can be stratified into groups that predict 8 week mortality ranging from 16-71% and 3 year overall survival ranging from 3-40%.

The goal of intensive chemotherapy for AML is achievement of a complete remission (CR), typically after 1 to 2 cycles of therapy. While achievement of CR has been shown to translate into longer survival compared to not achieving a CR, newer hypomethylating agents lead to hematologic improvement and even CR in some patients after several courses of therapy with less treatment related toxicity [5]. The question then arises in the elderly population whether rapid achievement of CR is necessary or therapies that prevent leukemia acceleration and reduce the burden of disease over time may be more beneficial.

To answer this question will require better understanding of the bone marrow micro-environment in AML patients. Prolonged cytopenias leading to infectious complications and bleeding is a common cause of death in AML, and the prolonged cytopenias seen with the slower response to hypomethylating agents must be considered when choosing this therapy over intensive chemotherapy. Novel therapies that improve cytopenias while at the same time reducing the burden of leukemia would be clearly beneficial in the elderly patients.

Over the last decade drug development and clinical trials in elderly AML have focused on less-intensive therapies that have the potential of achieving complete remission while preserving quality of life. These options include, among others, clofarabine, farnesyl transferase inhibitors, FMS-like tyrosine kinase 3 (FLT-3) inhibitors and sapacitabine. Despite the development of these new agents, there have been no approvals of new drugs for the treatment of AML in elderly patients by the United States FDA, highlighting the difficulty of obtaining a true survival benefit in this population.

Consolidation therapy, aimed at preserving remission, is paramount to treatment of AML upon achievement of a CR. Allogeneic hematopoietic cell transplantation (HCT) and chemotherapy consolidation are two therapeutic options to prevent relapse in younger patients. Unfortunately, both options are associated with significant morbidity in older AML patients without a clear track record of success. Non-myeloablative conditioning regimens have been developed to reduce toxicity and allow the use of HCT in older patients, but infections, graft-versus-host disease, and disease relapse remain common issues. However, non-myeloablative conditioning regimens have been used in well-selected older patients who can tolerate it with impressive long-term survival [6].

Recently, there has been significant excitement over the use of immune therapies in AML. The goal is to enhance immune cells and redirect the patient's own immune system to target leukemic cells. Several immunotherapeutic strategies are currently being in evaluated in proof of concept in clinical trials. The current armamentarium of immune approaches in acute leukemia includes bispecific T cell engager (BITE) antibodies, chimeric antigen receptors T-cells and check-point inhibitors [7]. Although these agents are early in development in AML, they provide hope for a novel, reduced toxicity anti-leukemia induction therapy or a unique method of delivering durable consolidation therapy for older adults.

Care for the older patient with AML remains challenging. However, careful selection of fit older patients who may benefit from intensive induction therapy and use of less toxic agents in others allows for successful treatment of some older patients with AML. Improving understanding of the molecular basis of AML and its sensitivity to immunotherapy offers hope for a more tailored and tolerable approach.
